# Adolescents who engage exclusively in healthy weight control behaviors: Who are they?

**DOI:** 10.1186/s12966-016-0328-3

**Published:** 2016-01-15

**Authors:** Amy M. Lampard, Richard F. Maclehose, Marla E. Eisenberg, Nicole I. Larson, Kirsten K. Davison, Dianne Neumark-Sztainer

**Affiliations:** School of Psychology and Speech Pathology, Curtin University, Kent St, Bentley, WA Australia; Department of Nutrition, Harvard School of Public Health, 665 Huntington Ave, Boston, MA 02115 USA; Division of Epidemiology and Community Health, School of Public Health, University of Minnesota, 1300 South Second Street, Suite 300, Minneapolis, MN 55454 USA; Division of General Pediatrics and Adolescent Health, Department of Pediatrics, University of Minnesota, 717 Delaware Street SE, 3rd Floor, Minneapolis, MN 55414 USA

**Keywords:** Adolescence, Health behavior, Physical activity, Eating behavior, Body dissatisfaction, Weight control behavior

## Abstract

**Background:**

Little is known about the exclusive adoption of healthy weight control behaviors in the absence of unhealthy weight control behaviors among adolescents. The current study aimed to determine (i) the prevalence of the exclusive adoption of healthy weight control behaviors, (ii) the pattern of eating behaviors and physical activity reported by those engaging exclusively in healthy weight control behaviors, and (iii) the socio-demographic and psychosocial factors associated with the exclusive use of healthy weight control behaviors among adolescents.

**Methods:**

In a large and diverse population-based sample of US adolescents (*N* = 2793) who participated in EAT 2010 (Eating and Activity in Teens) the current study examined the exclusive use of healthy weight control behaviors, which included healthy eating behaviors (eating more fruits and vegetables, eating less high-fat foods, eating less sweets, drinking less soda, and being aware of portion sizes) and engaging in physical activity for the purpose of weight management. Data were analyzed using multinomial logistic regression in STATA.

**Results:**

Overall, 24.0 % of girls and 29.2 % of boys exclusively used healthy weight control behaviors in the absence of unhealthy weight control behaviors. The exclusive use of healthy weight control behaviors was more prevalent among girls who were not overweight (27.5 %) as compared to girls who were overweight (21.0 %) or obese (17.5 %), controlling for age, socio-economic status, and ethnicity/race. In addition, the exclusive use of healthy weight control behaviors was more prevalent among girls and boys who had lower body dissatisfaction, higher self-esteem and lower depressive symptoms.

**Conclusion:**

Findings indicate that psychosocial health and body satisfaction may be important targets for promoting the exclusive use of healthy weight control behaviors among adolescents.

## Background

Adolescents face substantial pressure from peers, family, and the broader social and media environment to control their weight [1, 2]. The cross-national 2001–2002 Health Behaviour in School-aged Children (HSBC) study found that a high proportion of adolescents aged 13 to 15 years reported engaging in weight control behaviors in the past 12 months; specifically, across Belgium, Canada, Estonia, Finland, Greece, Latvia, Poland, and the US, the use of weight control behaviors ranged from 40 to 60 % among non-overweight girls, 10–34 % among non-overweight boys, 63–84 % among overweight girls, and 31–61 % among overweight boys [3]. Adolescents may adopt a range of weight control behaviors with the potential for positive or negative health consequences, depending on the type of weight control behaviors used. The HSBC study found that, among US adolescents attempting to lose weight, the vast majority reported engaging in exercise (93 % non-overweight girls; 90 % overweight girls; 97 % non-overweight boys; 95 % overweight boys) and eating more fruit and/or vegetables (62 % non-overweight girls; 70 % overweight girls; 66 % non-overweight boys; 54 % overweight boys) for weight control purposes [3]. In addition, the 2010 National Youth Physical Activity and Nutrition Study, a nationally representative sample of US adolescents in grades 9 through 12, found that 35 % of adolescents overall reported eating more fruit or vegetables in the past 30 days to lose weight or avoid gaining weight [4]. These examples of healthy weight control behaviors not only play a role in the prevention of excess weight gain but also in reducing future risk for chronic diseases such as heart disease, stroke, and diabetes [5–8].

While many adolescents engage in healthy weight control behaviors, unhealthy weight control behaviors are also prevalent among youth, including fasting, skipping meals, taking diet pills, and self-induced vomiting. Severe dieting (i.e., scoring in the top tertile on a measure of meal skipping, calorie counting, and food restriction) has been associated with a greater risk of eating disorder onset among adolescents [9]. Another recent nationally representative survey of US high school students in 2013 found that 63 % of girls and 33 % of boys reported trying to lose weight; overall, 13 % reported fasting, 5 % reported using diet pills, powders, or liquids, and 4 % reported self-induced vomiting or laxative use for the purpose of weight control [10].

Clearly, youth may engage in both healthy and unhealthy weight control behaviors but to the best of our knowledge, research has not yet examined the proportion of youth who engage *solely* in healthy weight control behaviors. Given the negative consequences of unhealthy weight control behaviors, a healthy pattern of behavior among adolescents involves the regular use of health-enhancing behaviors (i.e., healthy weight control behaviors) in the *absence* of health-compromising behaviors (i.e., unhealthy weight control behaviors). It is important to examine the prevalence and correlates of the exclusive use of healthy weight control behaviors, as the use of unhealthy weight control behaviors in combination with healthy weight control behaviors may (i) indicate the presence of unhealthy cognitions regarding the importance of weight and weight control, and (ii) place an individual at risk for eating disorder onset, health complications, or weight gain over time [11]. In addition, as unhealthy weight control behaviors are prevalent among adolescents, the prevalence of healthy weight control behaviors alone may be low, and this information is needed to inform public health communication.

Research is therefore needed to determine the prevalence of healthy weight control behaviors in the absence of unhealthy weight control practices and the profile of such behaviors used by those who exclusively engage in healthy weight control behaviors (i.e., type of healthy weight control behavior and number of healthy weight control behaviors). Further, to encourage adolescents to engage solely in healthy weight control behaviors, it is important to understand the psychosocial factors that may help or hinder adolescents to adopt such behaviors, yet such factors are currently unknown. Identifying the socio-demographic and psychosocial factors associated with the exclusive use of healthy weight control behaviors may inform efforts to promote healthy weight control behaviors among adolescents.

The current study therefore aimed to determine, in a large and diverse population-based sample of adolescents, (i) the prevalence of healthy weight control behaviors (i.e., eating more fruits and vegetables, eating less high-fat foods, eating less sweets, drinking less soda, being aware of portion sizes, and engaging in physical activity) in the absence of unhealthy weight control behaviors, (ii) the pattern of behavior reported by those engaging exclusively in healthy weight control behaviors, and (iii) the socio-demographic and psychosocial factors associated with the exclusive use of healthy weight control behaviors. Previous research has established links between overweight status, poor psychosocial health and the use of unhealthy weight control behaviors among adolescents [12–15]. It was therefore hypothesized that non-overweight adolescents and adolescents with more positive body image and psychosocial health (i.e., low levels of dissatisfaction with body fat, low levels of dissatisfaction with body build, high self-esteem, or low depressive symptoms) would have an increased likelihood of engaging exclusively in healthy weight control behaviors.

## Methods

### Participants and procedures

EAT 2010 (Eating and Activity in Teens; [16, 17]) examined eating behaviors, physical activity, weight status, and weight control behaviors in a population-based study of adolescents. Participants were students (*n* = 2793; 53.2 % girls) enrolled at 20 public middle (46.1 % in grades 6–8) and high (53.9 % in grades 9–12) schools in the Minneapolis/St Paul metropolitan area during the 2009–2010 academic year. The mean age of participants was 14.4 years (SD = 2.0). Racial/ethnic background was as follows: 18.9 % white, 29.0 % African-American or black, 19.9 % Asian-American, 16.9 % Hispanic, 3.7 % Native American, and 11.6 % mixed or other.

Trained research staff administered the EAT 2010 survey during health, physical education and science classes. Surveys were completed within the classroom setting and height and weight were measured in a private area. Adolescent and parental consent was obtained for 96.3 % of those attending school during survey administration. Following survey completion, participants received a $10 gift card. Study procedures were approved by the University of Minnesota’s Institutional Review Board Human Subjects Committee and by school district research boards.

### Measures

The EAT 2010 survey consisted of 235 items. Procedures used to generate the survey are outlined in detail elsewhere [18]. The EAT 2010 survey was pilot-tested with a sample of 129 middle and high school students (mixed gender; 70 % non-Hispanic white). The pilot-sample completed the survey on two occasions, one week apart. Test-retest reliability from this pilot-testing phase is reported below.

### Weight status

Height was measured without shoes to the nearest 0.1 cm and weight was measured without shoes or outerwear to the nearest 0.1 kg. Age- and sex-specific body mass index (BMI) percentiles were used to classify participants’ weight status (not overweight, overweight, obese) in accordance with CDC growth charts [19]; 19.5 % of girls and 16.0 % of boys were classified as overweight (≥85^th^, <95^th^ BMI percentile) and an additional 19.2 % of girls and 25.9 % of boys were classified as obese (≥95^th^ BMI percentile).

### Healthy weight control behaviors

Healthy weight control behaviors were assessed with the question, “How often have you done each of the following things in order to lose weight or keep from gaining weight during the past year”?, (i) exercise, (ii) ate more fruits and vegetables, (iii) ate less high-fat foods, (iv) ate less sweets, (v) drank less soda pop (not including diet pop), (vi) watched my portion sizes (serving sizes). Items were rated on a four point scale (“never”, “rarely”, “sometimes”, or “on a regular basis”). Participants reporting regular use of any of these behaviors were categorized as engaging in healthy weight control behaviors (test-retest agreement = 82.8 %).

### Unhealthy weight control behaviors

Unhealthy weight control behaviors were assessed with the question: “Have you done any of the following things in order to lose weight or keep from gaining weight during the past year”? (yes/no for each method). Behaviors assessed included the use of fasting, eating very little food, using a food substitute (i.e., powder or a special drink), skipping meals, smoking cigarettes, diet pills, self-induced vomiting, laxative use, or diuretic use for the purpose of weight control. Reporting the use of any of these weight control behaviors was categorized as engaging in unhealthy weight control behaviors (test-retest agreement = 85 %).

### Socio-demographic variables

Ethnicity/race was assessed with the item, “Do you think of yourself as… (i) white, (ii) black or African-American, (iii) Hispanic or Latino, (iv) Asian-American, (v) Native Hawaiian or other Pacific Islander, (vi) American Indian or Native American, or (vii) other”, and respondents were instructed to mark all categories that applied. Due to small numbers, those participants indicating Native Hawaiian, American Indian, or other ethnicity/race were all included in the “Other” category. A socio-economic status (SES) indicator was formed using an algorithm composed of parent education, eligibility for public assistance, eligibility for subsidized school meals, and parent employment, as described elsewhere [20].

### Psychosocial variables

Psychosocial variables assessed in the current study included body fat dissatisfaction, body build dissatisfaction, self-esteem, and depressive symptoms. Body dissatisfaction variables were assessed using a revised version of the Body Shape Satisfaction scale [21]. Body fat dissatisfaction was assessed with a seven item scale. Participants rated their satisfaction with body weight, body shape, waist, hips, thighs, stomach, and overall body fat on a five point scale ranging from “very dissatisfied” (1) to “very satisfied” (5). Items were reversed scored and summed to form a body fat dissatisfaction subscale, with higher scores indicating greater body fat dissatisfaction (range = 7 to 35; Cronbach’s alpha = .94; test-retest *r* = .73).

Body build dissatisfaction was assessed with a four item scale. Participants rated their satisfaction with their body build, shoulders, muscles, and chest on a five point scale ranging from “very dissatisfied” (1) to “very satisfied” (5). Items were reversed scored and summed, with higher scores indicating greater body build dissatisfaction (range = 4 to 20; Cronbach’s alpha = .89; test-retest *r* = .60).

Self-esteem was assessed using the six item Rosenberg Self-esteem Scale [22]. Items were rated on a four point scale, ranging from “strongly disagree” (1) to “strongly agree” (4). Higher scores represent higher self-esteem (scale range = 6 to 24; Cronbach’s alpha = .77; test-retest *r* = .69).

Depressive symptoms were assessed using the six item depression scale developed by Kandel and Davies [23]. Items assessed how often the respondent felt troubled by the following symptoms over the past 12 months: feeling tired; difficulty sleeping; feeling unhappy, sad or depressed; feelings of hopelessness; feeling nervous or tense; and worry. Items were rated on a three point scale, ranging from “not at all” (1) to “very much” (3). Higher scores indicate greater depressive symptoms (scale range = 6 to 18; Cronbach’s alpha = .83; test-retest *r* = .75).

### Data analysis

Participants were categorized into one of four weight control behavior categories: (i) *no weight control behavior* (reported engaging in no healthy weight control behaviors and no unhealthy weight control behaviors), (ii) *healthy weight control behaviors only* (reported engaging in one or more healthy weight control behaviors and no unhealthy weight control behaviors), (iii) *unhealthy weight control behaviors only* (reported engaging in one or more unhealthy weight control and no healthy weight control behaviors), or (iv) *mixed behavior* (reported engaging in both healthy weight control behaviors and unhealthy weight control behaviors).

The prevalence of each weight control behavior category was examined for girls and boys separately, stratified by weight status (not overweight, overweight, and obese). To further explore the behavior of those who reported only healthy weight control behaviors, the prevalence of each type of healthy weight control behavior and the total number of healthy weight control behaviors reported by those adolescents in this group was examined.

To estimate the association between socio-demographic factors (ethnicity/race, SES, weight status) and weight control behavior category, multinomial logistic regression models were fit in STATA adjusting for age (in years) with all socio-demographic and control variables (race, age, SES, and weight category) entered simultaneously. To determine the association between psychosocial variables (body fat dissatisfaction, body build dissatisfaction self-esteem, and depressive symptoms) and weight control behavior category, separate multinomial regression models were estimated, also adjusting for race, SES, weight status and age (in years). For these analyses, continuous independent variables (body fat dissatisfaction, body build dissatisfaction, self-esteem, and depressive symptoms) were split by tertile into low, medium, and high categories. Adjusted probabilities of each outcome were estimated from each of the multinomial regression models. These adjusted probabilities were used to compute pairwise comparisons between each level of the independent variable.

## Results

### Prevalence of weight control behaviors

The prevalence of each weight control behavior category stratified by adolescent weight status and sex is reported in Table 1. Of note, 24.0 % of girls and 29.2 % of boys reported the exclusive use of healthy weight control behaviors. Girls in the obese weight category reported the lowest prevalence of exclusive use of healthy weight control behavior (17.2 %) and the highest prevalence of mixed healthy and unhealthy weight control behavior (55.1 %).

**Table 1:** Observed prevalence of weight control behaviors stratified by weight status in girls and boys

		Weight status		
	Overall % (n)	Not overweight % (n)	Overweight % (n)	Obese % (n)
Girls				
No weight control	25.6 (380)	33.8 (307)	15.5 (45)	9.8 (28)
Healthy only	24.0 (356)	27.1 (246)	21.0 (61)	17.2 (49)
Unhealthy only	16.4 (244)	14.3 (130)	21.7 (63)	17.9 (51)
Mixed healthy and unhealthy	34.0 (504)	24.9 (226)	41.7 (121)	55.1 (157)
Boys				
No weight control	32.6 (424)	44.2 (333)	18.7 (39)	15.4 (52)
Healthy only	29.2 (379)	31.9 (240)	27.8 (58)	24.0 (81)
Unhealthy only	13.0 (169)	9.2 (69)	18.7 (39)	18.1 (61)
Mixed healthy and unhealthy	25.2 (328)	14.7 (111)	34.9 (73)	42.6 (144)

Note: Not overweight, <85^th^ BMI percentile; Overweight, ≥85th, <95^th^ BMI percentile; Obese, ≥95^th^ BMI percentile

The profile of weight control practices (i.e., exercise; increased consumption of fruit and vegetables; decreased consumption of sweets, soda, or high fat food; and managing portion sizes) reported by those exclusively using healthy weight control behaviors was further examined. Across all weight categories, the majority of adolescents exclusively using healthy weight control behaviors reported engaging in only one (44.9 % girls; 48.0 % boys) or two (24.7 % girls; 28.5 % boys) of the practices assessed (Table 2). Overall, among adolescents solely using healthy weight control behaviors, exercise was the most commonly reported behavior (54.1 % girls; 72.6 % boys), followed by increased consumption of fruit and vegetables (54.1 % girls; 41.0 % boys) and decreased consumption of soda (41.2 % girls; 33.2 % boys; Table 2). This pattern was consistent across those in the “not overweight” and overweight weight status categories (Table 2). Among girls in the obese weight category, increased consumption of fruit and vegetables was the most commonly reported behavior. Among boys in the obese weight category, exercise was the most commonly reported behavior, but in contrast to adolescents in the not overweight or overweight categories, decreased consumption of soda was more common than increased consumption of fruit and vegetables.

**Table 2:** Profile of weight control behavior use among those adolescents engaging solely in healthy behaviors to lose or maintain weight in the absence of unhealthy weight control behaviors, stratified by weight status and sex

		Weight status		
	Overall % (n)	Not overweight % (n)	Overweight % (n)	Obese % (n)
Girls				
Prevalence of specific types of healthy weight control behavior				
Exercise	54.1 (192)	55.5 (136)	60.7 (37)	38.8 (19)
Ate fruit and vegetables	54.1 (192)	52.0 (128)	52.5 (32)	66.7 (32)
Drank less soda	41.2 (145)	38.3 (93)	50.8 (31)	43.8 (21)
Ate less sweets	28.1 (99)	24.2 (59)	43.3 (26)	28.6 (14)
Ate less fat	26.7 (94)	22.5 (55)	42.6 (26)	27.7 (13)
Watched my portion size	18.4 (65)	16.3 (40)	27.9 (17)	16.7 (8)
Number of healthy weight control behaviors				
1	44.9 (160)	48.0 (118)	36.1 (22)	40.8 (20)
2	24.7 (88)	25.2 (62)	18.0 (11)	30.6 (15)
3	10.4 (37)	10.6 (26)	11.5 (7)	8.2 (4)
4	9.6 (34)	7.3 (18)	14.8 (9)	14.3 (7)
5	5.1 (18)	5.3 (13)	6.5 (4)	2.0 (1)
6	5.3 (19)	3.7 (9)	13.1 (8)	4.1 (2)
Boys				
Prevalence of specific types of healthy weight control behavior				
Exercise	72.6 (275)	75.0 (180)	77.6 (45)	61.7 (50)
Ate fruit and vegetables	41.0 (155)	43.9 (105)	41.4 (24)	32.1 (26)
Drank less soda	33.2 (122)	29.6 (68)	35.1 (20)	42.0 (34)
Ate less sweets	24.9 (94)	18.1 (43)	31.0 (18)	40.7 (33)
Ate less fat	17.3 (65)	16.5 (39)	20.7 (12)	17.3 (14)
Watched my portion size	11.7 (44)	7.6 (18)	17.2 (10)	19.8 (16)
Number of healthy weight control behaviors				
1	48.0 (182)	50.0 (120)	50.0 (29)	40.7 (33)
2	28.5 (108)	28.8 (69)	22.4 (13)	32.1 (26)
3	10.6 (40)	11.3 (27)	5.2 (3)	12.4 (10)
4	5.5 (21)	4.2 (10)	8.6 (5)	7.4 (6)
5	4.0 (15)	4.2 (10)	5.2 (3)	2.5 (2)
6	3.4 (13)	1.7 (4)	8.6 (5)	4.9 (4)

Note: Not overweight, <85^th^ BMI percentile; Overweight, ≥85th, <95^th^ BMI percentile; Obese, ≥95^th^ BMI percentile

### Associations of socio-demographic factors and weight status with weight control behavior type

Associations of socio-demographic factors and weight status with weight control behavior type were estimated for girls and boys (Table 3). Among girls, those who were not overweight/obese were significantly more likely to engage exclusively in healthy weight control behaviors (27.5 %) than those who were classified as overweight (21.0 %) or obese (17.5 %), adjusting for SES, age, and ethnicity/race. Also in girls, those in the middle and the middle-high SES groups were more likely (32.1 and 32.7 % respectively) than those in the low and low-middle groups (21.0 and 20.2 % respectively) to engage exclusively in healthy weight control behaviors, adjusting for weight status, age, and ethnicity/race. Some differences were observed across ethnicity/race groups; Hispanic (29.2 %), Caucasian (28.0 %), and African American (25.6 %) girls engaged exclusively in healthy weight control behaviors with the highest frequency while Asian American (19.1 %) girls were least likely to engage in healthy weight control behaviors. In contrast, among boys, weight status and ethnicity/race were unrelated to the probability of engaging exclusively in healthy weight control behaviors. In addition, among boys, the highest SES category (43.8 %) engaged in significantly more healthy weight control behaviors than the low (23.4 %), low-middle (30.3 %) and middle (30.0 %) SES groups.

**Table 3:** Predicted probabilities (95 % confidence interval) for engaging in weight control behavior types by ethnicity/race, socioeconomic status, and weight status in girls and boys

	Healthy WCB only	Comparison weight control behavior groups		
		No WCB	Unhealthy WCB only	Mixed WCB
Girls				
Weight status				
Not overweight	27.5 (24.5, 30.4)^a^	34.4 (31.2, 37.5)^a^	13.8 (11.6, 16.1)^a^	24.3 (21.5, 27.1)^a^
Overweight	21.0 (16.3, 25.7)^b^	15.0 (10.9, 19.1)^b^	22.2 (17.4, 27.0)^b^	41.8 (36.0, 47.5)^b^
Obese	17.5 (13.1, 22.0)^b^	9.0 (5.7, 12.3)^c^	17.8 (13.4, 22.3)^ab^	55.7 (49.8, 61.5)^c^
Socioeconomic status				
Low	21.0 (17.8, 24.3)^a^	25.9 (22.5, 29.3)^b^	19.1 (16.0, 22.1)^c^	34.0 (30.4, 37.6)^a^
Low-middle	20.2 (15.8, 24.6)^a^	28.8 (24.0, 33.7)^b^	16.9 (12.8, 21.0)^bc^	34.1 (29.0, 39.2)^a^
Middle	32.1 (26.3, 38.0)^b^	19.3 (14.5, 24.1)^a^	12.2 (8.0, 16.4)^ab^	36.4 (30.5, 42.3)^a^
Middle-high	32.7 (25.4, 39.9)^b^	24.2 (17.9, 30.5)^ab^	13.6 (8.1, 19.2)^abc^	29.5 (22.6, 36.5)^a^
High	26.5 (17.1, 35.9)^ab^	29.9 (20.6, 39.1)^b^	8.8 (2.1, 15.6)^a^	34.7 (24.6, 44.8)^a^
Ethnicity/race				
Caucasian	28.0 (22.3, 33.7)^c^	24.9 (19.5, 30.4)^a^	11.3 (6.8, 15.7)^a^	35.8 (29.6, 42.0)^bc^
African/American	25.6 (21.3, 29.8)^bc^	31.0 (26.6, 35.5)^a^	16.0 (12.6, 19.4)^a^	27.4 (23.3, 31.5)^a^
Hispanic	29.2 (23.4, 34.9)^c^	25.8 (20.4, 31.1)^a^	14.7 (10.4, 18.9)^a^	30.4 (24.9, 35.9)^ab^
Asian American	19.1 (14.5, 23.6)^a^	15.1 (11.1, 19.0)^b^	23.8 (18.8, 28.8)^b^	42.0 (36.3, 47.8)^c^
Other	19.5 (14.5, 24.4)^ab^	29.7 (24.1, 35.4)^a^	13.9 (9.5, 18.2)^a^	36.9 (31.1, 42.7)^bc^
Boys				
Weight status				
Not overweight	31.6 (28.2, 35.0)^a^	43.7 (40.1, 47.4)^a^	9.4 (7.2, 11.5)^a^	15.3 (12.7, 18.0)^a^
Overweight	28.9 (22.5, 35.3)^a^	19.7 (14.0, 25.3)^b^	17.3 (12.0, 22.6)^b^	34.1 (27.4, 40.8)^b^
Obese	25.7 (20.9, 30.6)^a^	16.1 (12.0, 20.2)^b^	15.6 (11.7, 19.6)^b^	42.5 (37.1, 47.9)^b^
Socioeconomic status				
Low	23.4 (19.3, 27.6)^a^	32.9 (28.4, 37.3)^a^	14.6 (11.3, 17.9)^a^	29.1 (24.9, 33.2)^c^
Low-middle	30.3 (25.0, 35.7)^b^	33.2 (27.9, 38.5)^a^	13.4 (9.5, 17.3)^a^	23.1 (18.4, 27.8)^abc^
Middle	30.0 (24.1, 35.9)^ab^	30.7 (25.0, 36.4)^a^	10.4 (6.5, 14.3)^a^	28.9 (23.3, 34.5)^bc^
Middle-high	34.0 (26.8, 41.3)^bc^	38.6 (31.6, 45.6)^a^	9.1 (4.5, 13.6)^a^	18.3 (12.4, 24.2)^a^
High	43.8 (34.3, 53.2)^c^	28.4 (20.6, 36.3)^a^	8.4 (2.8, 14.0)^a^	19.4 (11.7, 27.1)^ab^
Ethnicity/race				
Caucasian	29.1 (23.5, 34.6)^a^	36.9 (31.2, 42.6)^a^	9.7 (5.6, 13.7)^a^	24.3 (18.8, 29.9)^a^
African/American	30.5 (25.7, 35.3)^a^	30.2 (25.6, 34.8)^a^	13.2 (9.7, 16.7)^a^	26.1 (21.7, 30.5)^a^
Hispanic	31.8 (25.3, 38.3)^a^	33.2 (26.8, 39.6)^a^	11.9 (7.6, 16.1)^a^	23.1 (17.8, 28.4)^a^
Asian American	26.8 (21.1, 32.5)^a^	29.8 (24.0, 35.6)^a^	14.8 (10.4, 19.2)^a^	28.7 (23.3, 34.0)^a^
Other	30.0 (22.9, 37.0)^a^	35.7 (28.5, 42.9)^a^	10.6 (6.1, 15.2)^a^	23.8 (17.6, 29.9)^a^

Model adjusts for age in years, with all variables entered simultaneously

*WCB* weight control behavior

The presence of different superscript letters denotes statistically significant differences (*p* <.05) between predicted probabilities. Estimates sharing a superscript are not significantly different at *p* <.05

### Associations of psychosocial factors with weight control behavior type

Associations between psychosocial factors and weight control behavior type adjusted for socio-demographic factors and weight status were estimated for girls and boys (Table 4). A similar pattern of results was observed in both girls and boys for all psychosocial factors. Body fat dissatisfaction and body build dissatisfaction were significantly and inversely associated with the exclusive use of healthy weight control behaviors, even after controlling for weight status. Specifically, in girls, those with low body fat dissatisfaction or body build dissatisfaction were significantly more likely to engage exclusively in healthy weight control behaviors (33.3 and 31.0 % respectively) than those with medium body fat dissatisfaction or body build dissatisfaction (25.6 and 23.8 % respectively), who were in turn significantly more likely to engage exclusively in healthy weight control behavior than those with high body fat or body build dissatisfaction (17.2 and 18.5 % respectively). Similarly, in boys, those with low or medium body fat dissatisfaction or body build dissatisfaction were significantly more likely to engage exclusively in healthy weight control behaviors (36.0 and 35.4 % respectively for low; 30.4 and 31.5 % for medium) than those with high body fat dissatisfaction or body build dissatisfaction (21.6 and 20.4 % respectively).

**Table 4:** Predicted probabilities (95 % confidence interval) for engaging in each weight control behavior by psychosocial factors in girls and boys

	Healthy WCB only	Comparison weight control behavior groups		
		No WCB	Unhealthy WCB only	Mixed WCB
Girls				
Body fat dissatisfaction				
Low	33.3 (28.7, 37.9)^a^	34.8 (30.4, 39.2)^a^	12.6 (9.3, 15.9)^a^	19.3 (15.3, 23.3)^a^
Medium	25.6 (21.7, 29.5)^b^	25.4 (21.7, 29.2)^b^	15.5 (12.2, 18.7)^a^	33.5 (29.3, 37.7)^b^
High	17.2 (13.7, 20.7)^c^	15.1 (11.7, 18.6)^c^	21.4 (17.7, 25.1)^b^	46.3 (41.8, 50.8)^c^
Body build dissatisfaction				
Low	31.0 (26.6, 35.5)^a^	29.3 (25.2, 33.4)^a^	10.1 (7.1, 13.1)^a^	29.6 (25.1, 34.1)^a^
Medium	23.8 (20.5, 22.5)^b^	26.8 (23.4, 30.2)^a^	16.5 (13.6, 19.4)^b^	32.9 (29.3, 36.4)^a^
High	18.5 (14.4, 22.5)^c^	17.5 (13.5, 21.5)^b^	23.3 (19.0, 27.6)^c^	40.8 (35.9, 45.6)^b^
Self-esteem				
High	31.6 (27.2, 36.1)^a^	34.2 (29.9, 38.5)^a^	8.1 (5.4, 10.8)^a^	26.1 (21.9, 30.3)^a^
Medium	26.0 (22.4, 29.7)^a^	24.7 (21.2, 28.2)^b^	15.2 (12.2, 18.2)^b^	34.1 (30.2, 37.9)^b^
Low	16.0 (12.6, 19.4)^b^	16.3 (12.9, 19.7)^c^	25.8 (21.7, 29.8)^c^	41.9 (37.5, 46.4)^c^
Depressive symptoms				
Low	33.5 (28.6, 38.4)^a^	32.7 (28.0, 37.4)^a^	9.4 (6.3, 12.5)^a^	24.3 (20.0, 28.7)^a^
Medium	26.0 (22.2, 29.8)^b^	27.0 (23.3, 30.7)^a^	15.0 (11.9, 18.2)^b^	32.0 (28.0, 36.0)^b^
High	17.4 (14.2, 20.5)^c^	19.1 (15.9, 22.3)^b^	21.7 (18.4, 25.0)^c^	41.9 (37.9, 45.8)^c^
Boys				
Body fat dissatisfaction				
Low	36.0 (31.3, 40.7)^a^	36.2 (31.8, 40.6)^a^	8.7 (5.9, 11.6)^a^	19.1 (15.0, 23.1)^a^
Medium	30.4 (26.1, 34.6)^a^	35.1 (30.8, 39.4)^a^	12.8 (9.7, 15.9)^ab^	21.7 (18.0, 25.4)^a^
High	21.6 (16.6, 26.5)^b^	24.1 (18.8, 29.4)^b^	16.9 (12.6, 21.2)^b^	37.4 (31.9, 42.8)^b^
Body build dissatisfaction				
Low	35.4 (30.3, 40.6)^a^	33.1 (28.3, 37.8)^a^	9.0 (5.9, 12.2)^a^	22.5 (17.9, 27.0)^a^
Medium	31.5 (27.6, 35.4)^a^	33.2 (29.4, 37.0)^a^	12.0 (9.2, 14.7)^ab^	23.3 (19.9, 26.8)^a^
High	20.4 (15.9, 24.9)^b^	32.8 (27.6, 37.9)^a^	16.3 (12.4, 20.3)^b^	30.5 (25.8, 35.2)^b^
Self-esteem				
High	39.2 (34.6, 43.8)^a^	36.1 (31.9, 40.4)^b^	6.4 (4.0, 8.8)^a^	18.3 (14.6, 22.0)^b^
Medium	27.8 (23.6, 32.0)^b^	33.3 (29.1, 37.6)^ab^	11.4 (8.5, 14.4)^b^	27.4 (23.5, 31.4)^a^
Low	18.9 (14.4, 23.4)^c^	27.7 (22.7, 32.7)^a^	21.3 (16.8, 25.8)^c^	32.1 (27.2, 37.0)^a^
Depressive symptoms				
Low	31.7 (27.8, 35.7)^a^	37.6 (33.7, 41.5)^b^	10.4 (7.8, 13.0)^a^	20.3 (16.9, 23.6)^a^
Medium	31.1 (26.6, 35.7)^a^	31.9 (27.5, 36.4)^ab^	12.0 (8.8, 15.2)^ab^	24.9 (20.8, 29.1)^a^
High	23.8 (18.9, 28.8)^b^	25.5 (20.5, 30.4)^a^	16.0 (11.8, 20.1)^b^	34.7 (29.6, 39.9)^b^

Models adjust for age in years, socio-economic status, ethnicity/race and weight status

*WCB* weight control behavior

The presence of different superscript letters denotes statistically significant differences (*p* <.05) between predicted probabilities. Estimates sharing a superscript are not significantly different at *p* <.05

For both girls and boys, self-esteem and depressive symptoms were also significantly associated with the exclusive use of healthy weight control behaviors (Table 4). In both sexes, those with high self-esteem were significantly more likely to engage exclusively in healthy weight control behaviors (31.6 % among girls and 39.2 % among boys) than those with low self-esteem (16.0 % among girls and 18.9 % among boys). Similarly, those with low depressive symptoms were significantly more likely to engage exclusively in healthy weight control behaviors (33.5 % among girls and 31.7 % among boys) than those with high depressive symptoms (17.4 % among girls and 23.8 % among boys).

## Discussion

Arguably, a healthy behavior profile for adolescents includes engagement in healthy weight control behaviors (e.g., increasing fruit and vegetable consumption, eating less high fat food, and regulating portion sizes), such that these behaviors are adopted on a regular basis and point to a healthy lifestyle pattern. In addition, a healthy behavior profile includes the absence of any unhealthy weight control behaviors, including fasting, meal skipping, diet pill use, or purging behaviors. Such behaviors, even when adopted infrequently, signal willingness to compromise one’s overall health and well-being for a perceived short-term weight control benefit.

Using this framework, the results of this study indicate that approximately one-quarter of adolescents engage exclusively in regular healthy weight control behaviors. Among this group, the majority used only one or two health behavior strategies, with physical activity being the most commonly reported behavior. Previous studies have reported the prevalence of healthy weight control behaviors among adolescents without considering the simultaneous use of unhealthy weight control behavior and thereby overestimate the prevalence of optimal health behavior use among adolescents. In the current study, while over half of the sample reported engaging in regular healthy weight control behavior (58.0 % of girls and 54.4 % of boys), only some of these participants (41.4 % of the girls and 53.6 % of the boys) additionally abstained from unhealthy weight control behavior. Therefore, the profile of adolescent healthy weight control behavior becomes more concerning when the simultaneous use of unhealthy weight and shape control behaviors is also considered. Since the co-occurrence of healthy and unhealthy weight control behavior was prevalent in the current study, public health campaigns that aim to promote healthy weight control behaviors among adolescents may need to simultaneously communicate the importance of avoiding unhealthy weight control behaviors. To achieve this, it may be beneficial to promote health behaviors, such as physical activity and fruit and vegetable consumption, in the absence of weight-related implications, and rather encourage their use for the purpose of overall health promotion.

Additionally, the current study found that adolescent girls and boys who reported lower body fat or body build dissatisfaction, higher self-esteem, or lower depressive symptoms were more likely to report a healthier behavior profile than those adolescents who reported high body fat or body build dissatisfaction, low self-esteem or high depressive symptoms. These results therefore indicate that those with high body dissatisfaction and poorer psychosocial health are at risk of failing to adopt a healthy behavior profile. These adolescents may need particular support to exclusively adopt healthy weight control behaviors. This finding builds on a vast literature linking these psychosocial factors with dieting, disordered eating, and eating disorders [24, 25], further supporting the need to address these factors in school settings to improve adolescent health.

The finding that both girls and boys with lower body fat and body build dissatisfaction were more likely to engage exclusively in healthy weight control behaviors than those with higher body fat and body build dissatisfaction is particularly noteworthy. This result is in line with previous research linking low body dissatisfaction with engagement in positive health behaviors. Prospective research has found that adolescents with lower body dissatisfaction report greater engagement in physical activity over a 5-year period than adolescents with higher body dissatisfaction, controlling for initial weight [26]. In addition, in a study focused on overweight adolescent girls, those with lower body dissatisfaction were found to gain less weight over a 5-year period than those with higher body dissatisfaction, adjusting for initial weight [27]. In combination with these previous findings, the results from this study run counter to claims that feeling dissatisfied with body weight or shape may be necessary to motivate health-promoting behaviors. The current study therefore provides further impetus to the need to reduce body dissatisfaction among adolescents and indicates that promoting a positive body image may be an important component of obesity prevention and health promotion campaigns [28].

This study also identified a link between weight status and health behaviors. Specifically, girls who were not overweight were more likely to engage exclusively in healthy weight control behaviors than those in the overweight or obese weight categories. A similar trend was observed in boys, although this was not statistically significant. It is important to note that while girls in the obese category reported less exclusive use of healthy weight control behavior, they did not report lower weight control behavior overall. In fact, girls in the obese weight category were less likely to report no weight control behaviour and more likely to report a mix of healthy and unhealthy weight control behaviour than those in the not overweight or overweight categories. As the vast majority of girls in the obese weight category reported engaging in at least one of the weight control behaviors assessed in the current study, results suggest that interventions targeted to this group do not need to motivate weight control behavior per se, but more specifically encourage engagement in healthy weight control behavior in the absence of unhealthy weight control behavior. These findings suggest a need to work closely with girls with higher BMI values, who are most likely to be experiencing pressures to lose weight and are utilizing unhealthy weight control practices in addition to healthy weight control behaviors.

Strengths of the current study include the use of a large population-based sample of adolescents and the objective measurement of height and weight. A number of measurement issues may be considered limitations in the study design. First, retrospective self-report of behaviors was a particular limitation for the measurement of weight control behaviors. Second, given the dichotomous response format used to identify the use of unhealthy weight control behaviors, the frequency with which participants in this group engaged in unhealthy weight control behavior cannot be determined; some participants in this group may have rarely engaged in unhealthy weight control. Third, those in the “no weight control behavior” group may include those who have used alternative weight control methods that were not specifically assessed in this study, or those who engaged in healthy behaviors but not for purposes of weight control. The current study is also limited by a cross-sectional design and thus conclusions cannot be made regarding the temporality of associations between psychosocial health and healthy weight control behaviors. While it is likely that psychosocial health (i.e., low body dissatisfaction, high self-esteem, and low depressive symptoms) supports the exclusive use of positive health behaviors, it is also possible that engagement in healthy weight control behaviors may in itself promote more positive psychosocial health. Indeed, it may be most likely that the relationship between these variables is bi-directional in nature, with both psychosocial health and healthy weight control behaviors feeding back into each other over time.

It is important to note that the current study is based on the framework that the adoption of healthy weight control behaviors in the absence of unhealthy weight control behaviors is the optimal health behavior profile for adolescents. In support of this argument is evidence linking unhealthy weight control behaviors to negative outcomes, including the onset of more severe disordered eating behaviors and eating disorders [9]. In addition, a large body of research has linked fruit and vegetable consumption, physical activity and a lower consumption of sugar-sweetened beverages to reduced risk for negative health outcomes, including cardiovascular disease and weight gain [29–31]. However, longitudinal investigations are needed to determine the effect of each weight control behavior profile on long-term health outcomes to identify a healthy behavior profile for adolescents.

## Conclusion

This population-based survey found that only approximately one quarter of adolescents engaged exclusively and regularly in healthy weight control behaviors. Observed associations between psychosocial health and health behavior indicate that promoting positive psychosocial health and body satisfaction may be an important component of campaigns designed to promote healthy weight control behaviors. In addition, campaigns may need to simultaneously emphasize the importance of both engaging in healthy weight control behaviors and not engaging in unhealthy weight control behaviors, particularly for adolescents in the overweight or obese weight categories. Further, it may be beneficial to promote these healthy behaviors (e.g., exercise and fruit and vegetable consumption) in the absence of a weight control context, such that all are encouraged to enjoy the health benefits of these behaviors, regardless of weight.
